# Bitter Melon (*Momordica Charantia*), a Nutraceutical Approach for Cancer Prevention and Therapy

**DOI:** 10.3390/cancers12082064

**Published:** 2020-07-27

**Authors:** Subhayan Sur, Ratna B. Ray

**Affiliations:** 1 Department of Pathology, Saint Louis University School of Medicine, St. Louis, MO 63104, USA; Subhayan.sur@health.slu.edu; 2 Cancer Center, Saint Louis University School of Medicine, St. Louis, MO 63104, USA

**Keywords:** medicinal plant, bitter melon (*Momordica charantia*), Cucurbitaceae, signal transduction, cancer prevention, cancer therapy

## Abstract

Cancer is the second leading cause of death worldwide. Many dietary plant products show promising anticancer effects. Bitter melon or bitter gourd (*Momordica charantia*) is a nutrient-rich medicinal plant cultivated in tropical and subtropical regions of many countries. Traditionally, bitter melon is used as a folk medicine and contains many bioactive components including triterpenoids, triterpene glycoside, phenolic acids, flavonoids, lectins, sterols and proteins that show potential anticancer activity without significant side effects. The preventive and therapeutic effects of crude extract or isolated components are studied in cell line-based models and animal models of multiple types of cancer. In the present review, we summarize recent progress in testing the cancer preventive and therapeutic activity of bitter melon with a focus on underlying molecular mechanisms. The crude extract and its components prevent many types of cancers by enhancing reactive oxygen species generation; inhibiting cancer cell cycle, cell signaling, cancer stem cells, glucose and lipid metabolism, invasion, metastasis, hypoxia, and angiogenesis; inducing apoptosis and autophagy cell death, and enhancing the immune defense. Thus, bitter melon may serve as a promising cancer preventive and therapeutic agent.

## 1. Introduction

Cancer is characterized by uncontrolled cell proliferation achieved by dynamic changes in the nuclear genome [1,2,3]. Studies identify several risk factors that influence uncontrolled cell proliferation such as: intrinsic risk arising from spontaneous mutations in DNA; external factors including carcinogens, viruses, xenobiotic and lifestyle factors like smoking, alcohol abuse, nutrient intake, physical activity; and endogenous factors that are related to the individual’s immune system, pattern of metabolism, DNA damage response and hormone levels [4]. In the USA, the predicted cancer incidences in the year 2020 will be around 18 lakhs, which is the equivalent of approximately 4950 new cases each day. The estimated deaths from cancer in 2020 will be around 6 lakhs corresponding to more than 1600 deaths per day [5]. Although prostate, lung and colorectal cancers are the most common cancers in men (account for 43% of all cases) and breast, lung, and colorectal cancers the most common in women (50% of all), the incidence of other cancers in the kidney, pancreas, liver, oral cavity and pharynx (head and neck) and skin continues to increase [5]. Despite significant improvement in therapies in the past few years, cancer is the second leading cause of death, and population-based studies project a dramatic increase in new cancer cases to more than 22 million globally by 2030 [6]. Thus, prevention and development of specific therapeutic agents will be key ways to manage the disease. Studies suggested that prevention can be achieved by reducing risk from external factors and lifestyle factors as well as by early detection [3]. In case of therapy, primary tumors can generally be removed through surgery but in some cases, surgery is difficult and not valid for sub-clinical metastases and cannot eliminate the cancer cells, resulting in relapse. In the case of targeted therapy, over the years more potent agents have been developed with less toxic effects, proper dosing and combination treatment protocols. However, these methods exert side effects, are sometimes expensive and one of the main problems is the eventual resistance of cancer cells to treatment [7].

A recent WHO report suggested that around 80% of the world population uses traditional herbal medicine for primary healthcare needs [8]. Some registered drugs such as vinca alkaloids (vinblastine, vincristine, vindesine, vinorelbine), taxanes (paclitaxel, docetaxel), podophyllotoxin and its derivations (topothecan, irinothecan) and anthracyclines (doxorubicin, daunorubicin, epirubicin, idarubicin) are derived from natural sources [7]. Several epidemiological studies suggest important roles of fruits and vegetables in reducing cancer risk [9]. This could be due to the cumulative effect of many bioactive phytochemicals, vitamins, minerals, proteins and fibers in the fruits and vegetables. Many plant products, either whole extract or bioactive components, are able to inhibit carcinogenesis, at least in animal models. There are many ongoing clinical trials to test the safety and efficacy of natural agents in preventing or treating cancer.

In the present review, we have focused on updated information for bitter melon (*Momordica charantia*) on cancer prevention and therapy and its underlying mechanisms. Bitter melon, bitter gourd, balsam pear or karela belongs to the family Cucurbitaceae and is widely cultivated in Asia, Africa and South America. The medicinal value of bitter melon has been reported from ancient times for the remedy of diseases like toothache, diarrhea, furuncle and diabetes [10]. The beneficial effects of bitter melon crude extract or isolated compounds are associated with lowering diabetes and lipidemia, anti-bacterial, antifungal and anti-HIV activities [9,11,12]. Promising anticancer effects of bitter melon were seen in different in vitro and in vivo studies [9,11,12,13,14]. We summarize here the molecular mechanisms of cancer prevention and therapy by bitter melon. Thus, this review has wide implications for the management of disease that may help in progression towards clinical studies. 

## 2. Bitter Melon and Its Constituents

**Figure 1:**
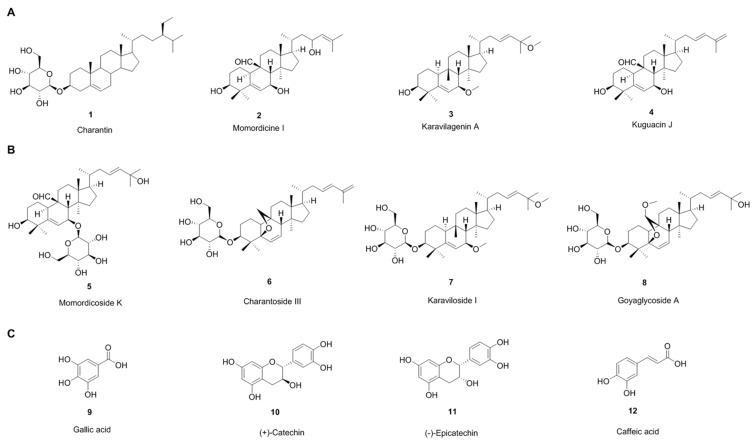
Chemical structure of some of the major components of bitter melon. (**A**): cucurbitane-type triterpenoids, (**B**): cucurbitane-type triterpene glycosides, and (**C**): phenolic compounds.

Bitter melon is a bitter tasting herbaceous plant cultivated in tropical and subtropical regions of many countries. Traditionally, bitter melon is used in different countries as a folk medicine. The fruits are also used as a side dish in southeast Asia. Bitter melon tea, which is known as gohyah or herbal tea, is made from dried slices and has been used for medicinal purposes [10]. Bitter melon has the highest nutritive values among cucurbits and contains over 30 medicinal products, including carbohydrates, proteins, fibers, vitamins (C, A, E, B1, B2, B3, and B9 as folate), and minerals (potassium, calcium, zinc, magnesium, phosphorous and iron) [11,12,13]. The biological activity of bitter melon depends on its major chemical constituents, including cucurbitane-type triterpenoids, cucurbitane-type triterpene glycosides, phenolic acids, flavonoids, essential oils, fatty acids, amino acids, lectins, sterols and saponin (goyasaponins I, II and III) constituents and some proteins present in fruits, seeds, roots, leaves and vines [11]. Cucurbitane-type triterpenoids are the most prevalent chemical constituents. The bitterness is the consequence of cucurbitane-type triterpenoids: (momordicines I (Figure 1A, #2) and II and triterpene glycosides: momordicosides K (Figure 1B, #5), and L [12]. Researchers have developed different extraction procedures to isolate pure compounds or plant extracts with different solvents like water, methanol, ethanol, n-butanol and acetone. Organic solvents are better for the extraction of phenolic acids and flavonoids [15,16]. There are different varieties of bitter melon, different origins, harvest times, and depending on those parameters, the proportion of chemical constituents varies. Different major constituents found in different varieties and different parts of the plants are summarized below [11,12,17].

### 2.1. Cucurbitane-Type Triterpenoids

Charantin (Figure 1A, #1), momordicine I (Figure 1A, #2), II and III, karavilagenin A (Figure 1A, #3), B, C, D and E, kuguacins A–S (Figure 1A, #4) are major components. Other components include: 23-dimethoxy-cucurbita-5, 24-dien-19-al, 19-epoxycucurbita-6, (23E)-3β-hydroxy-,7β, 25-dimethoxycucurbita-5, 23-diene-19-ol, 19-epoxy-19, 25-dimethoxycucurbita-6, 23-diene-3β-ol, (19R, 23E)-5β, 19-epoxy-19-methoxy cucurbita-6, 23-diene-3β, 25-diol, 25-dihydroxy-7β-methoxy cucurbita-5, 23(E)-diene, 3β-hydroxy-7β, 25-dimethoxy cucurbita-5, 23(E)-diene, 3β, 7β, 25-trihydroxy cucurbita-5, 23(E)-diene-19-al, 5β, 19-epoxycucurbita-6, 23(E)-diene-3β, 19, 25-triol, 5β, 19-epoxy-19-methoxycucurbita-6,23(E)-diene-3β, 25-diol; 3β, 25-dihydroxy-5β,19-epoxycucurbita-6, 23(E)-diene; 19(R)-methoxy-5β,19-epoxycucurbita-6, 23-diene-3β, 25-diol, 5β, 19-epoxycucurbita-6, 23(E)-diene-3β, 25-diol; 3β, 7β-dihydroxy-25-methoxy cucurbita-5, 23(E)-diene-19-al; 23(E)-25-methoxy cucurbita-23-ene-3β, 7β-diol, 23(E)-cucurbita-5, 23, 25-triene-3β, 7β-diol, 23(E)-25-dihydroxy cucurbita-5, 23-diene-3, 7-dione, 23(E)-cucurbita-5, 23, 25-triene-3, 7-dione, 23(E)-5β, 19-epoxycucurbita-6, 23-diene-3β, 25-diol, 23(E)-5β, 19-epoxy-25-methoxy cucurbita-6, 23-diene-3β-ol; cucurbita-5, 23(E)-diene-3β, 7β, 25-triol, 3β-acetoxy-7β-methoxy cucurbita-5, 23(E)-diene-25-ol, cucurbita-5(10), 6, 23(E)-triene-3β, 25-diol, cucurbita-5, 24-diene-3, 7, 23-trione; (19R, 23E)-5β, 19-epoxy-19-methoxy cucurbita-6, 23,25-triene-3β-ol, (23E)-3β-hydroxy-7β-methoxycucurbita-5, 23, 25-triene-19-ol.

### 2.2. Cucurbitane-Type Triterpene Glycosides

These include momordicosides (A–E, F1, F2, G, I, K, L, M, N, O, Q, R, S and T) (Figure 1B, #5), charantosides I–VIII (Figure 1B, #6), karaviloside (I–XI) (Figure 1B, #7), goyaglycoside- (a–h) (Figure 1B, #8), kuguaglycoside, 3-O-β-D-allopyranosyl, 7β, 25-dihydroxycucurbita-5, 23(E)-diene-19-al; 3-O-β-D-allopyranosyl, 7β, 25-dihydroxy cucurbita-5(6), 23(E)-diene-19-al, 3-O-β-D-allopyranosyl, 25-methoxy cucurbita-5(6), 23(E)-diene-19-ol, 24(*R*)-stigmastan-3β, 5α, 6β-triol-25-ene 3-*O*-β-glucopyranoside. 

### 2.3. Phenolic Acids and Flavonoids

These include galic acid (Figure 1C, #9), tannic acid, (+)-catechin (Figure 1C, #10), epicatechin (Figure 1C, #11), caffeic acid (Figure 1C, #12), p-coumaric, gentisic acid, and chlorogenic acid.

### 2.4. Proteins

Several proteins were identified and characterized from bitter melon extract. These include momordica antiviral protein 30kD (MAP30), α- and β-momocharin, 14-kDa Ribonucleases (RNase MC2) and marmorin. 

## 3. The Activity of Bitter Melon on Cancers

**Figure 2:**
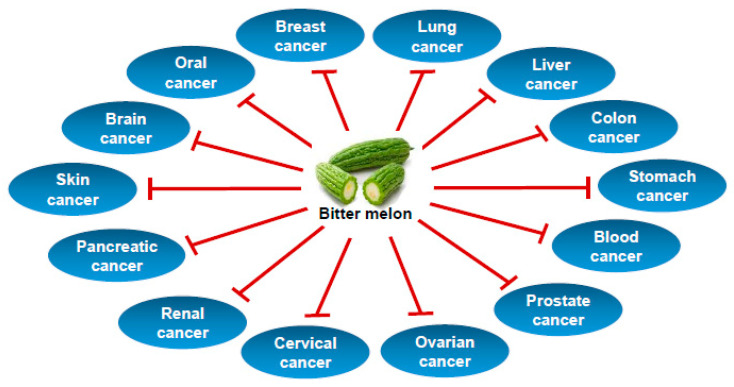
Types of cancer prevented by bitter melon.

Bitter melon extract and its active ingredients were studied in laboratory cancer cell line-based models and pre-clinical animal models, whereas clinical studies on cancers are lacking. The preventive studies were conducted in animal models of blood, breast, colon, head and neck, liver, prostate, skin and stomach cancers using mainly crude extract of bitter melon prepared by water, methanol or ethanol. Therapeutic studies using crude extracts or isolated compounds have been conducted in in vitro and in vivo models of blood, brain, breast, colon, gastric, head and neck, kidney, liver, lung, ovary, pancreas, prostate, skin and uterine cervical cancers. The effect of bitter melon on cancer chemoprevention and therapy are summarized in Table 1, Figure 2 and discussed below. 

### 3.1. Blood Cancer

The cancer preventive effect of crude bitter melon extract was first reported in a mouse model where the ammonium acetate precipitates of bitter melon water extract prevented tumor formation and enhanced immune function [22]. However, the crude extract showed minimum effect on normal human peripheral blood lymphocytes as compared to lymphocytes from patients with chronic or acute leukemia. Similarly, the bitter melon compound momordica antiviral protein 30kD (MAP30) significantly inhibited proliferation and induced apoptosis in the human acute myeloid leukemia (AML) cell line HL-60, THP-1 cells and patient AML cells in a dose- and time-dependent manner [18]. Fractions from seed extract, namely, Mc-1, Mc-2, Mc-3 and Mc-2Ac induced differentiation of leukemia cell HL60 in a dose-dependent manner [19]. In another study, (9Z,11E,13E)-15,16-dihydroxy-9,11,13-octadecatrienoic acid (15,16-dihydroxy α-eleostearic acid), which is a major component in seeds, induced apoptosis in HL60 cells [20]. The α-eleostearic acid isolated from ethanol extraction of seed inhibits proliferation of leukemia cell lines ED and Su9T01, whereas a minimal effect was reported on peripheral blood mononuclear cells [21].

### 3.2. Breast Cancer

Both preventive and therapeutic studies were conducted on breast cancer models. The water extract of fruit inhibited proliferation and induced apoptosis in breast cancer cells MCF-7 and MDA-MB-231 in a time- and dose-dependent manner with 80% reduction in cell viability [27]. Importantly, the extract showed no cytotoxic effect on primary mammary epithelial cells (HMEC) even after treatment for five days. Like the water extract, the isolated compound MAP30 inhibited MDA-MB-231 cells in in vitro and in vivo xenograft models in SCID mice [70]. Continuous administration of water extract (0.5%) through drinking water prevented spontaneous mammary tumor development in SHN virgin mice with no adverse side effects [33]. In syngeneic (mouse breast cancer cells 4T1 and E0771) and xenograft (human breast cancer cell MDA-MB-231) mouse models, oral feeding of the extract (30% *v/v*) through drinking water inhibited tumor growth, induced autophagy and reduced cholesterol esterification [28,29]. Bitter melon extract showed better effects on triple negative breast cancer cells in mouse models as compared to ER-positive breast cancer cells.

### 3.3. Colon Cancer

Bitter melon seed oil in diets regressed azoxymethane (AOM)-induced colon cancer incidence and multiplicity in a dose-dependent manner in male F344 rats [36]. Free fatty acid and 9-cis, 11-trans, 13-trans-conjugated linolenic acid isolated from bitter melon seed oil reduces the cell viability of Caco-2cells [38]. In addition, bitter melon seed extract in water, ethanol, or ethanol: water (1:1) showed cytotoxic effects on human colon tumor 116 cells [39]. However, the water extract showed the best effect on the cells. In addition, methanol extract of whole fruit inhibited proliferation, colony formation, sphere formation and induced autophagy in HT-29 and SW480 cells [34]. The extract prepared from whole skin showed a lower effect on the cell lines as compared to whole fruit extract. Neither of the extracts displayed any cytotoxic effect on noncancerous human foreskin fibroblast (HFF). The extract also increased doxorubicin sensitivity in colon cancer cells [35]. Konishi et al. identified the active component 1-monopalmitin from the methanol extract that inhibits P-glycoprotein in human epithelial colorectal adenocarcinoma cells Caco2 [71]. The α-eleostearic acid also inhibited growth of HT29 colon carcinoma cells [20]. 

### 3.4. Gastric Cancer

Short term and long term administration of fruit extract (2.5% and 5%) inhibited benzo(a)pyrene [B(a)P]-induced forestomach carcinogenesis in Swiss albino mice [66]. Long-term treatment showed better preventive effects in mice. The methanol extract of leaf exhibited therapeutic effects on gastric adenocarcinoma cells AGS, [68]. Bitter melon protein compounds (Fractioned I–III) isolated by high-speed counter-current chromatography inhibited human gastric cancer cell line SGC-7901 [67]. The fraction II showed the best anticancer activity.

### 3.5. Head and Neck Cancer

This category includes cancers of the tongue, oral cavity, nasal cavity, paranasal sinuses, saliva glands, larynx and pharynx. The bitter melon extract exhibited potential cytotoxic effect in Cal27, JHU029 and JHU022 cells in a time- and dose-dependent manner [44]. The anticancer effect was associated with inhibition of cell proliferation, induction of apoptosis, inhibition of c-Met signaling and reduction in glycolysis and lipid metabolism [40,44]. Oral administration of the extract (30% *v/v*) prevented xenograft and syngenic tumor growth by reducing cell proliferation and inducing apoptosis in mice [43,44]. Further, in the syngenic model, the extract reduced infiltrating regulatory T (Treg) cell populations in the tumors and in spleens [43]. Subsequent studies showed continuous oral administration of water extract through drinking water (30% *v/v*) prevented 4-nitroquinoline 1-oxide (4-NQO)-induced mouse tongue squamous cell carcinoma development at the pre-neoplastic stages through modulation in proliferation, ossification, metabolism and immune system [41]. In nasopharyngeal carcinoma cells, the Bitter melon component alpha-momorcharin (α-MMC) showed cytotoxic activity on CNE-1 and HONE1 cells, whereas minimal effect was seen in non-cancerous human nasopharyngeal epithelial cells NP69 [13].

### 3.6. Liver Cancer

Oral administration of methanol extract (40 mg/kg) at the pre- and post-initiation stages of carcinogenesis prevented hepatocellular carcinoma development induced by diethylnitrosamine (DENA) and carbon tetrachloride (CCl4) through modulation in expression of different genes associated with angiogenesis, proliferation, metastasis and apoptosis [48]. On the other hand, treatment with the fruit extract (5% *v/v*) for 48 h caused 63% cell death in HepG2 cells by inhibiting apolipoprotein B secretion and hepatic triglyceride synthesis [49]. The MAP30 and α-MMC isolated from seeds showed potential cytotoxic effects on HepG2 cells [47,51]. Map30 also inhibited HepG2 cell xenograft tumor growth in nude mice [47]. No side effect of MAP30 was seen in the animal model. Cucurbitane-type triterpene glycosides furpyronecucurbitane A, goyaglycoside I, charantagenin F and nine other compounds were examined for their anti-fibrosis activity against murine hepatic stellate cells (t-HSC/Cl-6) and anti-cancer activity against human hepatoma cells HepG2 and Hep3B [46]. Among the compounds, karaviloside-III showed the best inhibitory activity against t-HSC/Cl-6, Hep3B and HepG2 cell lines.

### 3.7. Lung Cancer

Water extract and methanol extract of bitter melon plant leaf showed cytotoxic effects on human non-small cell lung cells A549 and lung adenocarcinoma cells CL1 in a dose-dependent manner, whereas normal human embryonic kidney HEK293 cells and lung WI-38 cells are less susceptible [50,52]. The α-MMC and MAP30 suppressed proliferation and induced S-phase cell cycle arrest and apoptosis in A549 cells in a dose- and time-dependent manner [51]. The MAP30 showed more potent effects than α-MMC on the cells.

### 3.8. Pancreatic Cancer

Treatment with water extract of fruit inhibited cell proliferation and induced apoptosis in human pancreatic carcinoma cells BxPC-3, MiaPaCa-2, AsPC-1 and Capan-2 [55]. The extract inhibited proliferation and induced autophagy in gemcitabine-resistant AsPC-1 cells in a dose- and time-dependent manner [56]. The extract also inhibited CD44+/CD24+/EpCAMhigh pancreatic cancer stem cell (CSC) populations, CSC-associated markers SOX2, OCT4, NANOG and CD44 in-vitro and in-vivo, and increased sensitivity to gemcitabine [57]. In a xenograft model, the extract regressed tumor volume and inhibited the glucose and lactate transporters GLUT1 and MCT4 [58].

### 3.9. Prostate Cancer

Oral delivery of bitter melon fruit extract prevented the progression of prostatic intraepithelial neoplasia in TRAMP (transgenic adenocarcinoma of mouse prostate) mice by interfering with cell-cycle progression and proliferation [61]. Ethanol extract of leaves in the diet (1% and 5%) prevented PC3 cell xenograft tumor incidence without adverse effect on mouse body weight [59]. The same extract (0.1 and 1% in diet) increased animal survival and reduced PLS10 cell-mediated metastasis in nude mice [60]. Bitter melon extract induced more than 90% cell death in PC3 and LNCaP cells, whereas primary prostate epithelial cells exhibited very modest effects [61]. Another study reported that whole fruit water extract inhibits growth and induces G2-M phase cell cycle arrest of rat prostatic adenocarcinoma cells, in vitro [72]. The ethanol extract of leaves inhibited prostate cancer growth in in vitro and in vivo models [59,60]. Bitter melon compound MAP30 (1–20 µg/mL) reduced cell proliferation and induced apoptosis in human prostatic intraepithelial neoplasia (PIN) cells, PC-3 cells and LNCaP cells in a dose-dependent manner with no cytotoxic effect on normal prostate cells RWPE-1 [62]. Intraperitoneal administration of MAP30 also inhibited PC-3 xenograft tumor growth in nude mice.

### 3.10. Skin Cancer

Oral administration of the fruit extract prevented carcinogen-induced skin carcinogenesis in mice, increased survival, reduced lipid peroxidation, activated the liver enzymes glutathione-S-transferase, glutathione peroxidase and catalase, and reduced DNA damage in lymphocytes [65]. Similarly, pre-treatment or continuous local application of methanol extract of fruit and leaf (at doses of 500 and 1000 mg/kg body weight) significantly reduced dimethylbenz[a] anthracene (DMBA)/croton oil-induced skin papilloma formation, prevented micronucleus formation and chromosomal aberrations and increased survival in Swiss albino mice [63]. Cucurbitane-type triterpene glycosides compounds 1 and 2 isolated from methanol extract of fruit prevented DMBA and peroxynitrite-induced mouse skin carcinogenesis [64]. However, no further studies have been reported with these compounds. In a melanoma therapeutic model, 500 and 1000 mg/kg body weight dose of fruit and leaf extracts in 50% methanol reduced B6F10 xenograft tumor growth and increased survival of C57 B1 mice [63].

### 3.11. Other Cancers

Crude bitter melon extracts or isolated compounds showed potential anticancer effects on other cancers like adrenocortical cancer, glioma, ovarian cancer and uterine cervical cancers. The extract inhibited proliferation and induced apoptosis in human and mouse adrenocortical cancer cells, whereas extract from blueberry, zucchini, and acorn squash did not show any cytotoxic effect [45]. MAP30 inhibited cell proliferation, migration and invasion and induced apoptosis in the glioma cell lines U87 and U251 in a time- and dose-dependent manner [23]. The methanol extract of fruit inhibited cell proliferation and increased cisplatin sensitivity in the ovarian cancer cell lines A2780cp, A2780s, C13* and OV2008 [54]. No significant cytotoxicity of the extract was reported on immortalized human ovarian surface epithelial (HOSE 17-1) cells. Intraperitoneal injection of the extract regressed ES2 xenograft tumor growth and increased cisplatin sensitivity in nude mice. Similarly, the ethanol extract of leaves inhibited proliferation and induced sensitivity to the chemotherapeutic drugs vinblastine and paclitaxel in the human cervical cancer cell line KB-V1 in a dose-dependent manner [69]. The hexane and diethyl ether fractions from the extract showed the most potent effect. However, extracts from the fruits and tendrils showed no effect on these cells. Kuguacin J (#4) isolated a methanol extract of leaf and exhibited cytotoxicity and induced drug sensitivity on cervical cancer cells KB-VI and ovarian cancer cells SKOV3 [53,69]. Purified lectins momordin (MW: 24 kDa) and agglutinin (MW: 32kDa) inhibited Ehrlichascites tumor at an LD50 dose of 5 mg per kg body weight with no apparent animal toxicity [73].

## 4. Molecular Mechanism of Bitter Melon in Cancer Prevention and Therapy

**Figure 3:**
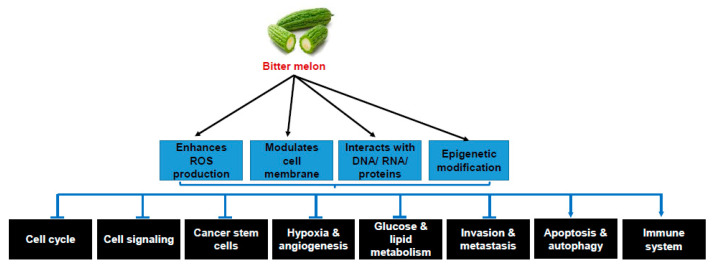
Molecular mechanisms of cancer prevention and therapy by bitter melon. Sharp arrow indicates activation/induction and blunt arrow indicates inhibition.

The biological activity of bitter melon depends on the cumulative effect of different bioactive components. The cancer preventive and therapeutic action of bitter melon crude extract/pure compounds depends on the time of administration, i.e., pre- or post-initiation stages of carcinogenesis, but the molecular mechanisms of prevention and therapy were found to be similar. The molecular mechanisms of the anticancer effects of bitter melon were extensively studied in in vitro cancer cell line models. Based on these studies, along with a few in vivo studies, the molecular mechanisms of bitter melon are discussed below and summarized in Table 2, Figure 3.

### 4.1. Generation of Reactive Oxygen Species, Anti-Inflammation and Carcinogen Elimination

Bitter melon crude extract and pure compounds enhanced cellular reactive oxygen species (ROS) generation, reduced inflammatory cytokines s100a9, IL23a, IL-1β, IL-6 and TNFα, and induced activity of different detoxification enzymes including glutathione-s-transferase, superoxide dismutase and catalasein in different cancers (Table 2). Since tumor cells have enhanced production of ROS, further increments of ROS levels along with the induction of detoxification enzymes prevent tumor initiation and progression and enhance stress-induced cell death. Many natural products exert similar mechanisms of chemoprevention [78]. Though acute inflammation is a primary response against pathogen or carcinogenic insult, chronic inflammation achieved by induction of the pro-inflammatory cytokines is one of the causes of carcinogenic transformation by increasing ROS level, inducing mutation, epithelial-to-mesenchymal transition (EMT), angiogenesis, and metastasis [79]. Inhibition of the pro-inflammatory cytokines by inhibitors or neutralizing antibodies shows promising results in different clinical trials [79]. On the other hand, detoxification enzymes including glutathione-s-transferase, superoxide dismutase and catalase act as the first line of defense system against oxidation and carcinogen metabolism, and thus prevent carcinogenesis initiation and progression [80,81]. Thus, bitter melon exerts potential preventive and therapeutic effect against several cancers.

### 4.2. Regulation of Cell Cycle

Cancer cells are characterized by unregulated cell cycle progression and defective cell cycle check points that contribute to uncontrolled proliferation, genetic instability and resistance to apoptotic cell death [82]. Cell cycle-specific qRT-PCR arrays and subsequent validation by western blot analysis revealed that bitter melon water extract inhibits cell cycle-promoting genes cyclin D1 and survivin and induces tumor suppressor gene p21 and p27 in head and neck cancer cells [44]. The water extract induced S or G2-M phase cell cycle arrest, inhibited expression of cyclin D1, cyclin E1 and cyclin B1 and enhanced p53, p21, and pChk1/2 in breast and prostate cancer cells [27,61]. Similar effects of crude extract and bitter melon components α-MMC, MAP30, kuguacin J (#4) and lectin were observed in other cancers (Table 2).

### 4.3. Modulation in Cell Signaling

During cancer progression, cancer cells manipulate several signaling pathways to favor unregulated proliferation, motility and survival [83]. Many of the signaling molecules have been investigated as a potential target for cancer therapy. Bitter melon extract inhibited the c-Met/Stat3/c-Myc/Mcl-1 axis in head and neck cancer [44]. The proto-oncogene MET encodes receptor tyrosine kinase c-Met that promotes tumor development and progression by regulating multiple downstream events including STAT3/c-Myc, PI3K/AKT, Ras/MAPK, JAK/STAT, SRC and Wnt/β-catenin [84]. Further, the extract activated AMP-activated protein kinase (AMPK) and inhibited the mTOR/p70S6K and/or the AKT/ERK/FOXM1 (Forkhead Box M1) signaling cascade in ovarian cancer [54]. Similarly, the crude extract modulated AMPK/mTOR and p38 MAPK signaling in breast cancer, colon cancer, and prostate cancer [28,34,61]. Bitter melon compounds α-eleostearic acid, 3β, 7β, 25-trihydroxycucurbita-5,23(E)-dien-19-al, lectin and RNase MC2 showed potential roles in regulating signaling events in different cancers (Table 2). Several studies suggest that c-Met, PI3K/AKT or p38 MAPK signaling are attractive targets for drug development to inhibit proliferation and resistance to apoptosis, and many such drugs showed promising effects in clinical trials against multiple cancers [84,85,86]. Thus, modulation of the signaling events by bitter melon may have importance in cancer prevention and therapy.

### 4.4. Induction of Apoptosis and Autophagy

Apoptosis and autophagy are considered as inter-connected pathways of cell death [87], and bitter melon triggers both the pathways to induce cancer cell death. Apoptosis is caspase-mediated programmed cell death and is activated in response to various stresses such as DNA damage, growth factor withdrawal and oxidative stress [87]. Generally, solid tumors lose the ability to undergo instantaneous and massive apoptosis, the so-called primary response that characterizes sensitive cells due to genetic mutations or alteration [88]. Induction of apoptosis is a required event for different classes of anticancer agents, and disruption of this mechanism can lead to broad drug resistance and sometimes non-specific side effects [88]. Bitter melon crude extract was found to enhance expression of pro-apoptotic Bax, Bak, Bid and p53, reduce anti-apoptotic Bcl2, activate caspase 3, 7, 9, cytochrome-c release, and induce PARP cleavage in prevention of various cancers (Table 2). Similarly, induction of apoptosis was seen by bitter melon compounds α, β- momorcharin, RNase MC2, 3β,7β,25-trihydroxycucurbita-5,23(E)-dien-19-al, MAP30, lectin and BG-4 in different cancers. Autophagy is a self-degradative process in response to various stresses, including nutrient deficiency, organelle damage, hypoxia, ROS generation, ER stress, and drug treatment [87]. Autophagy mechanisms in cancer are not clear: sometimes it is pro-tumorigenic, whereas sometimes it is beneficial for cancer prevention and excessive autophagy facilitates massive cell death [87,89]. Bitter melon extract induced autophagic cell death by converting LC3A to lipidated LC3B, increasing accumulation of p62, and enhancing expression of Beclin-1, ATG-7 and -12 (Table 2). Bitter melon lectin also plays a dual role by inducing either apoptosis or autophagy [13]. However, the mechanism of induction of autophagy or apoptosis in cancers following treatment with bitter melon is not known.

### 4.5. Inhibition of the Cancer Stem Cell Population

Cancer stem cells are small sub-populations of cells in heterogeneous tumors and give rise to a new tumor with the phenotype of the original one when transplanted into a host, undergo self-renewal, differentiation and contribute to chemotherapy or radiotherapy resistance, metastasis and tumor relapse [90,91]. CSCs can be detected by different markers including Sox2, Oct4, Nanog, CD24, CD44, CD133, CD90, EpCAM and ALDH in various tumors [90,92]. Targeting CSCs in combination with conventional therapy is suggested to be an important approach for chemotherapy, and a number of clinical trials are ongoing against different cancers [90]. Many natural phytochemicals exhibit anticancer properties by targeting the CSC population and its self-renewal [92]. Bitter melon water extract could inhibit CD44+/CD24+/EpCAMhigh CSC populations, decrease CSC markers SOX2, OCT4, NANOG and CD44 and enhance gemcitabine sensitivity in pancreatic cancer models [57]. Similarly, methanol extract of fruit inhibited sphere formation and expression of CSC marker DCLK1 and Lgr5 in colon cancer cells [34]. MAP30 reduced expression of the self-renewal Wnt pathway effector molecule β-Catenin and its target genes c-Myc and cyclin D1 in glioma and prostate cancer cells [23,62]. Thus, bitter melon may have potential therapeutic implications against different cancers through its action on CSCs.

### 4.6. Modulation in Glucose and Lipid Metabolism

Metabolic reprogramming is one of the hallmarks of cancer that favors rapid energy production, biosynthetic capabilities and therapy resistance. RNAseq analysis reveals down-regulation of key glycolysis and lipid metabolism genes in prevention of mouse tongue carcinogenesis by bitter melon water extract [40]. Subsequent analysis in head and neck cancer cells revealed down-regulation of key glycolysis genes SLC2A1 (Glut-1), PFKP, LDHA, PKM and PDK3, and reduction in pyruvate and lactate levels and glycolysis rates following treatment with the extract [40]. In lipid metabolism, the water extract inhibited expression of the fatty acid biogenesis genes ACLY, ACC1 and FASN and reduced levels of phosphatidylcholine (PC), phosphatidylethanolamine (PE) and plasmenylethanolamine (pPE) in head and neck cancer cells [40]. In a triple negative breast cancer (TNBC) model, the water extract reduced esterified cholesterol by inhibiting acyl-CoA: cholesterol acyltransferase 1 (ACAT-1) [29]. Subsequent studies showed reduced expression of lipid metabolism genes SREBP-1/2, FASN, LDLR and TIP47 as well as lipid droplet accumulation in the TNBC cells by the extract. Modulation of lipid metabolism by bitter melon induced ER-stress-mediated apoptotic cell death [29,40]. Treatment with water extract also reduced glucose transporter GLUT1 and lactate transporter MCT4 in in- vitro and in-vivo models of pancreatic cancer [58]. Thus, the modulation of metabolism is an important event of bitter melon-mediated cancer prevention and therapy.

### 4.7. Modulation in Immune System

Immune suppression is an important event in carcinogenesis. The bitter melon whole fruit water extract reduced FoxP3+ infiltrating regulatory T (Treg) cell populations in tumors and in spleens [43]. In addition, the extract reduced Th17 cell populations in tumors; however, there was no change in the Th1 and Th2 cell populations. Further, treatment with the extract enhanced natural killer (NK) cell-mediated cytotoxic effects in head and neck cancer cells [42]. However, the extract did not show any cytotoxic effect on the NK cells, but enhanced granzyme B accumulation, translocation/accumulation of CD107a/LAMP1 and expression of CD16 and NKp30. RNA sequence analysis revealed that the water extract significantly modulates the "immune system process" in the prevention of mouse tongue carcinogenesis [41]. The significantly down-regulated genes of this pathway were s100a9, IL23a, IL1β and the immune checkpoint gene PDCD1/PD1 in the bitter melon-treated group. Elevated expression of s100a9, IL23a, IL1β and PD1 were observed in several human malignancies. Pharmaceutical targeting of s100a9 or PD1 showed promising results in phase I–III clinical trials against different cancers [93,94,95]. Thus, bitter melon extract shows a potential role in cancer prevention and therapy.

### 4.8. Inhibition of Invasion, Metastasis, Hypoxia and Angiogenesis

Bitter melon water extract inhibited wound healing, migration and invasion in ovarian cancer cell line SKOV3 [54]. Methanol extract of bitter melon leaf inhibited migration and invasion and suppressed enzymatic activity of MMP-2 and MMP-9 in human lung adenocarcinoma CL1 cells [52]. Similarly, ethanol extract of leaf inhibited migration and invasion of rat prostate cancer cells (PLS10) by inhibiting MMP-2, MMP-9, urokinase plasminogen activator (uPA) and collagenase type IV activity and by inducing expression of TIMP2 [60]. The bitter melon component α-MMC reduced expression of hypoxia-inducible factor 1-alpha (HIF1α) and vascular endothelial growth factor (VEGF) in hypoxic nasopharyngeal carcinoma cells, and inhibited growth of human umbilical vein endothelial cells [77].

All together, bitter melon extract or pure compounds modulate multiple cellular events at a time to prevent cancer cell proliferation, survival and metastasis. How the extract or its compounds regulate different events simultaneously to exhibit anticancer effects is, however, unclear. The possible mechanisms of bitter melon and its compounds in this regard are discussed below.

### 4.9. How Does Bitter Melon Extract Enter into Cancer Cells?

To execute anticancer activity, bitter melon extract/compounds must interact with the cancer cell membrane and thereafter enter cancer cells. Little is known about this mechanism. In one study, it was evident that bitter melon water extract could inhibit expression of membrane lipid raft protein Flotilin and modulate its localization in head and neck cancer cells [40]. In the same study, bitter melon extracts reduced levels of cell membrane components phosphatidylcholine, phosphatidylethanolamine, and plasmenylethanolamine in head and neck cancer cells. This indicates that the extract might interact with the lipid bilayer and regulate cancer cell membrane integrity and permeability. Lipid rafts are also receptor-mediated cell signaling hubs. Thus, bitter melon-mediated modulation in different signaling events might be due to modulation of membrane lipid rafts. Lectin-type compounds are found to bind specifically to cell surface oligosaccharides and glycan, and are transported into cells [96,97]. Cancer cells modulate their membrane structure from normal cells in many ways. Among them, alteration in membrane oligosaccharides is observed predominantly in cancer cells [97]. Different types of lectins are present in bitter melon extract, which may act similarly to enter specifically into cancer cells and exhibit biological mechanisms including inhibition of ribosomes and induction of apoptotic and autophagic cell death [96,98]. Different triterpene glycosides bind to cell membranes, interact with membrane lipids and form glycoside–sterol complexes in the membrane, resulting in the formation of multimeric channels in sterol-containing lipid bilayers and increased permeability of membranes to ions and peptides [99]. Saponin types of compounds also have the ability to bind to the cell surface, to form pores in the membrane and disrupt the ionic balance in the membrane, resulting in cell lysis [100]. Similarly, flavonoids can easily bind to the cell surface, enter the cells and exhibit cytotoxic effects [101]. Thus, it seems that types of triterpene glycosides, saponins and flavonoids present in bitter melon may act by similar mechanisms to enter into cells and show anticancer effects. However, detailed studies are needed to know the exact mechanisms by which bitter melon extract modulates membrane integrity and thereafter enters into cells to show biological effects.

### 4.10. How Does Bitter Melon Regulate Gene Function?

Bitter melon extract and its compounds suppress the function of oncogenes and induce expression of tumor suppressive genes at a time to exhibit anticancer effect. Next generation RNAseq analysis showed that 4482 genes were differentially regulated in the prevention of mouse tongue cancer by bitter melon [41]. Subsequent analysis revealed that the genes significantly regulate multiple biological processes including “signal transduction,” “apoptosis process,” “metabolic process,” “cell adhesion,” “lipid metabolism,” “immune system process,” “angiogenesis,” “ossification,” and “G1/S transition of mitotic cell cycle.” An antibody array from bitter melon extract-treated breast cancer cells showed significant inhibition of survivin, XIAP, claspin and Bcl2 proteins and up-regulation of catalase, Bax and p27 proteins [27]. The underlying mechanism may include:

#### 4.10.1. Interaction with Cellular Macromolecules DNA, RNA and Proteins

Bitter melon components α-MMC and MAP30 have DNase activity and topological inactivation of DNA activity [102]. These two components were found to be potent inhibitors of protein synthesis due to their ribosome-specific N-glycosidase activity [102]. The RNases MC2, found in bitter melon seed, showed potent RNA-cleavage activity toward baker’s yeast tRNA, tumor cell rRNA, and an absolute specificity for uridine [75]. In the same study, RNase MC2 induced nuclear damage by karyorrhexis, chromatin condensation and DNA fragmentation, resulting in early/late apoptosis in the breast cancer cell line MCF7 [75]. Bitter melon lectins have type I and II ribosome inactivation activity [22,98]. Another study showed that a purified factor from bitter melon extract (molecular weight corresponding to 40 kDa) inhibits RNA and protein synthesis in intact tissue culture cells [103]. A protein component (molecular weight corresponding to 50–70 kDa) present in water extract showed non-competitive inhibition of guanylate cyclase [22]. Bitter melon extract exhibits P-glycoprotein inhibitory activity [37]. ABC transporter P-glycoprotein is highly expressed in tumor cell membranes and excretes hydrophobic drugs from the cells in an ATP-dependent manner, resulting drug resistance [37]. Bitter melon extract inhibits the activity of calcium-independent phospholipase A2 (iPLA2) in head and neck cancer cells [40]. iPLA2 is ubiquitously expressed in mammalian cells and participates in several biological processes including lipid metabolism, phospholipid remodeling, cell differentiation, maintenance of mitochondrial integrity, cell proliferation, signal transduction and cell death [40]. Studies suggest that flavonoids physically interact with DNA, RNA and protein molecules, thereby regulating transcription, translation, protein function and enzymatic activity [104,105]. The flavonoids form strong hydrogen bonds, enabling them to bind strongly with nucleic acids and proteins. Bitter melon extract contains several flavonoids; it seems that those components may act in a similar way. There is no study indicating how bitter melon components enter the nucleus. All the evidence indicates that bitter melon components enter cells and regulate the functions of DNA, RNA and protein in cancer prevention and therapy.

#### 4.10.2. Epigenetic Modification

Epigenetic regulation, including DNA methylation at CpG dinucleotide sequences, histone modifications such as methylation and acetylation, and non-coding RNA-mediating regulation, are reversible processes and play crucial roles in gene expression. Epigenetic changes are essential events that regulate activation of oncogenes and suppression of tumor suppressor genes, and are widely seen at early stages of carcinogenesis [106]. Many medicinal plant extracts and active components can reverse epigenetic modifications, thereby exhibiting anticancer properties [107]. The role of bitter melon in epigenetic modification is not well studied. Bitter melon extract contains many phytochemicals, particularly flavonoids. Flavonoids were found to alter epigenetic mechanisms in the restriction of cancer [108]. A bitter melon triterpenoid, 3β,7β,25-trihydroxycucurbita-5,23(E)-dien-19-al (TCD), inhibits histone deacetylases (HDAC1, HDAC2, HDAC3 and HDAC4) in the prevention of breast cancer cell growth [30]. Bitter melon MCP30 inhibits histone deacetylase-1 (HDAC-1) activity and promotes histone H3 and H4 acetylation in prostate cancer cells [62]. MAP30 induces p300, a histone acetyltransferase, and promotes histone H3 acetylation in leukemia cells [18]. Bitter melon fruit extract shows anti-inflammatory effects in human lung epithelial cells by upregulating micro RNAs miR-221 and miR-222 [109]. This indicates that regulation in gene expression by bitter melon might be due to epigenetic modification activity; however, detailed studies are needed to elucidate these mechanisms.

## 5. Conclusions

As discussed in this review, bitter melon is rich in many nutrients and active components including triterpenoids, triterpene glycoside, phenolic acids, flavonoids, lectins, sterols, proteins and saponins. The cancer preventive and therapeutic efficacy of bitter melon was extensively studied using crude extract in water, methanol and ethanol as solvents. Both crude extract and isolated compounds have potential cancer preventive and therapeutic effects to inhibit cancer cell proliferation, survival and metastasis against several cancers without any significant toxicity in normal cells. The anticancer effects are associated with ROS generation, activation of detoxification enzymes, inhibition of cancer stem cell populations and their self-renewal, inhibition of cell cycle, cell signaling, invasion, metastasis, hypoxia, angiogenesis, glucose and lipid metabolism, induction of apoptosis and autophagy and modulation in the immune system (Figure 3). Alterations in multiple cellular events may be achieved simultaneously due to modulation of membrane organization, interaction with DNA, RNA and proteins, and epigenetic modifications by bitter melon (Figure 3). Thus, it seems that bitter melon may improve cancer preventive machinery. On the other hand, the extracts or pure compounds may be used as therapeutic agents alongside conventional therapy for additional cancer treatment management. However, further evaluation of active components and in-depth mechanistic study in pre-clinical systems are needed, which may have importance for designing prospective studies for interventional therapies.

## 6. Future Directions

Bitter melon extract is considered as a popular health drink despite its bitter taste. Bitter melon is rich in nutrients and bio-active components. The crude extract and some isolated compounds have been studied against different cancers in cell culture and pre-clinical animal models; however, we still need to identify whether these isolated compounds possess similar effects to crude bitter melon extract or not. We also do not know whether mixture of some of those compounds will be more efficacious or not. More preclinical studies are needed for in-depth evaluation of therapeutic efficacy. Some ambiguities in different studies are present due to different methods of extraction, different varieties of fruits and different doses. There is a lack of information about metabolism and bioavailability of the compounds discovered. Further, there are limited studies using the same purified compounds in different cancer preclinical models, raising questions about the specificity of the active components. Preventive roles of bitter melon in many cancers are well-studied in pre-clinical models; however, anticancer studies using bitter melon as a combination with standard therapy are also limited. Thus, there is an opportunity to explore these research areas and carefully design the clinical studies to fight against cancer.

## Figures and Tables

**Table 1:** Roles of bitter melon in cancer prevention and therapy.

Cancer Model	Bitter Melon Extract/Compounds	Preventive and Therapeutic Effects	Ref.
Blood	Seed extract, water extract of fruit, MAP30 and α-eleostearic acid	Inhibited the proliferation of leukemia cells HL-60, THP-1, HL60 ED, Su9T01, HUT-102 and Jurkat and induced apoptosis. Inhibited in-vivo tumor formation in mice, increased survival and immune function.	[18,19,20,21,22]
Brain	MAP30, α, β momorcharin, charantagenins D, E, and sterol, 7-oxo-stigmasta-5,25-diene-3-O-β-d-glucopyranoside	Inhibited proliferation, migration, invasion and induced apoptosis in glioma cells	[23,24,25,26]
Breast	Water extract of fruit, dried extract and isolated compounds 3β,7β,25-trihydroxycucurbita-5,23(E)-dien-19-al (TCD), eleostearic acid, RNase MC2, MAP30	Inhibited breast cancer cells growth, induced apoptosis and autophagy. Inhibited syngenic tumor, xenograft tumor and spontaneous mammary tumorigenesis in SHN virgin mice.	[13,26,27,28,29,30,31,32,33]
Colon	Methanol extract of fruit, seed extract, seed oil, α-eleostearic acid, MAP30 and some isolated cucurbitane-type triterpene glycosides	Inhibited colon cancer cell proliferation, induced cell cycle arrest, apoptosis, autophagy, doxorubicin sensitivity and inhibited cancer stem cells. Prevented azoxymethane (AOM)-induced colon carcinogenesis in F344 rats.	[13,20,26,34,35,36,37,38,39]
Head and neck	Water extract of fruit	Inhibited oral cancer cell proliferation, metabolism, and induced apoptosis in oral cancer cells. Regressed oral cancer syngenic tumor, xenograft tumor and 4NQO-induced mouse tongue carcinogenesis.	[40,41,42,43,44]
Kidney	Water extract	Inhibited adrenocortical cancer cell proliferation, steroidogenesis and induced apoptosis.	[45]
Liver	Water extract of fruit, methanol extract and isolated compounds karaviloside III, MAP30, RNase MC2, lectin.	Inhibited murine hepatic stellate cells and human liver cancer cells. Prevented xenograft tumor growth in nude mice and DENA/CCl_4_ induced liver carcinogenesis in rats.	[13,46,47,48,49]
Lung	Water extract, methanol extract of leaf, MAP30 and α-MMC.	Inhibited proliferation, migration, invasion, and induced cell cycle arrest and apoptosis in human lung cancer cells.	[50,51,52]
Ovary	Water extract of fruit and kuguacin J	Inhibited growth, induced apoptosis and cisplatin sensitivity in human ovarian cancer *invitro* and *invivo* models.	[53,54]
Pancreas	Water extract of fruit	Prevented proliferation, metabolism and induced apoptosis in cancer cells and xenograft tumor.	[55,56,57,58]
Prostate	Water extract of fruit, leaf extract, kuguacin J, 30 kDa protein from seeds (MCP30)	Inhibited cell proliferation, cell cycle and metastasis in prostate cancer cells. Prevented xenograft tumor and spontaneous tumor in TRAMP mice.	[59,60,61,62]
Skin	Water extract of fruit, methanol extract of fruit and leaf, and cucurbitane-type triterpenes compounds from fruit	Prevented melanoma syngeneic tumor growth, DMBA/croton oil or DMBA/peroxynitrite induced skin carcinogenesis in mice.	[63,64,65]
Stomach	Fruit extract, methanol extract of leaf and fractioned proteins I–III	Showed anti-cancer activities in human gastric cancer cell lines. Prevented benzo(a)pyrene [B(a)P] induced forestomach papillomagenesis in mice.	[66,67,68]
Uterine cervix	Leaf extract and kuguacin J	Inhibited vinblastine and paclitaxel resistance in human cervical carcinoma cell line (KB-V1).	[69]

**Table 2:** Molecular mechanisms of bitter melon in cancer prevention and therapy.

Molecular Events	Bitter Melon Extract/Compound	Molecular Roles	Cancer Model	Reference
Reactive oxygen species (ROS) generation, anti-inflammation, carcinogen elimination	Fruit extract, triterpenoid (3β,7β,25-trihydroxycucurbita-5,23(E)-dien-19-al)	Induced ROS generation, activity of different detoxification enzymes including glutathione-s-transferase, superoxide dismutase and catalase, and reduced pro-inflammatory cytokines.	Head and neck cancer, lung cancer and breast cancer cells, alcohol-induced rat liver injury, 4NQO-induced mouse tongue cancer	[30,40,41,50,74]
Regulation in cell cycle	Fruit extract, α-MMC and MAP30, kuguacin J, lectin	Induced G2/M phase and S phase cell cycle arrest, inhibited cyclin D1, cyclin B1, cyclin E, survivin, Cdk2, Cdk4 and induced p21, p27, p53, pChk1/2	Breast cancer, prostate cancer, colon cancer, lung cancer, and head and neck cancer cells	[13,14,27,34,44,51,61]
Modulation in cell signalling	Crude extract, α-eleostearic acid, 3β, 7β, 25-trihydroxycucurbita-5,23(E)-dien-19-al, lectin, RNase MC2	Inhibited c-Met/Stat3/c-myc and Mcl-1 signalling, AKT/mTOR/p70S6K signalling, p38 MAPK signalling, AMPK signalling, AKT/ERK/FOXM1 signalling	Head and neck cancer, ovarian cancer, breast cancer, lung cancer, prostate cancer, nasopharyngeal cancer and pancreatic cancer cells	[14,44,54,75]
Induction of Apoptosis and autophagy	Crude extract, α, β- momorcharin, RNase MC2, 3β,7β,25-trihydroxycucurbita-5,23(E)-dien-19-al, MAP30, lectin, BG-4	Induced activation of caspases, pro-apoptotic genes, reduced anti-apoptotic genes, and induced PARP cleavage. Induced long chain 3 (LC3)-B and p62/SQSTM1 (p62), Beclin-1, ATG-7 and 12	Breast cancer, prostate cancer, head and neck cancer, colon cancer, lung cancer, pancreatic cancer, hepatocellular carcinoma, glioma, leukemia cells	[13,14,27,28,44,50,55,61,75,76]
Inhibition of cancer stem cell population	Fruit extract, MAP30	Inhibited cancer stem cells and stem cell markers SOX2, OCT4, NANOG and CD44, suppressed Wnt/β-catenin signalling.	Colon cancer, pancreatic cancer, prostate cancer and glioma cells	[23,34,57,62]
Inhibition of hypoxia and angiogenesis	α-MMC	Reduced HIF1α, VEGF, unfolded protein response (UPR), IRE-1,	Nasopharyngeal Carcinoma	[77]
Modulation in glucose and lipid metabolism	Crude extract	Inhibited key glycolysis and fatty acid metabolism genes, phospholipid synthesis and cholesterol esterification.	In-vivo and in-vitro model of head and neck cancer, breast cancer and pancreatic cancer	[29,40,58]
Modulation in immune system	Crude extract	Inhibited immune check point gene PD1, cytokines s100a9, IL23a, IL1β. Induced natural killer cell-mediated cytotoxicity. Inhibited Treg cell and Th17 cell population.	In-vivo and in-vitro model of head and neck cancer	[41,42,43]
Inhibition of invasion and metastasis	Crude extract, kuguacin J	Inhibited MMP9, MMP2, collagenase type IV activity, increased TIMP2	Lung adenocarcinoma cell, ovarian cancer cell, rat prostate cancer cells	[52,54,60]

## Abbreviations

ROS	Reactive oxygen species
αMMC	α-Momorcharin
MAP30	Momordica Antiviral Protein 30kD
iPLA2	calcium-independent phospholipase A2
DMBA	7, 12-Dimethylbenz(a)anthracene
4NQO	4-Nitroquinoline 1-oxide
ER	Endoplasmic Reticulum
c-Met	MET Proto-Oncogene
DENA	Diethylnitrosamine
CCl_4_	Carbon tetrachloride
AMPK	5′-AMP-Activated Protein Kinase
AKT	AKT Serine/Threonine Kinase
mTOR	Mechanistic Target of Rapamycin Kinase
TRAMP	Transgenic Adenocarcinoma of the Mouse Prostate
TNFα	Tumor Necrosis Factor α
IL23a	Interleukin 23 Subunit Alpha
IL1β	Interleukin 1 Beta
IL6	Interleukin 6
SOX2	SRY-Box transcription Factor 2
OCT4	Octamer-binding transcription factor 4
Cdk2/4	Cyclin Dependent Kinase 2/4
MAPK	Mitogen-Activated Protein Kinase
ERK	Extracellular signal-regulated kinase
FOXM1	Forkhead box protein M1
RAC	RacFamily Small GTPase
CDC42	Cell division control protein 42 homolog
PARP	Poly (ADP-ribose) polymerase
Bax	BCL2-Associated X, Apoptosis Regulator
Bak	Bcl-2 homologous antagonist/killer
Bid	BH3-Interacting Domain Death Agonist
Bcl2	BCL2 Apoptosis Regulator
LC3-B	Microtubule-Associated Protein 1 Light Chain 3 Beta
ATG-7/12	Autophagy-Related 7/12
MMP2/9	Matrix Metallopeptidase 2
TIMP2	TIMP Metallopeptidase Inhibitor 2
HIF1α	Hypoxia-inducible factor 1-alpha
VEGF	Vascular endothelial growth factor
UPR	Unfolded Protein Response
IRE-1	The serine/threonine-protein kinase/endoribonuclease inositol-requiring enzyme 1 α
GLUT-1	Glucose transporter 1
SLC2A1	Solute Carrier Family 2 Member 1
PFKP	Phosphofructokinase Platelet
MCT4	Monocarboxylate transporter 4
LDHA	Lactate dehydrogenase A
PKM	Pyruvate kinase isozymes M1/M2
PDK3	Pyruvate Dehydrogenase Kinase 3
ACLY	ATP Citrate Lyase
ACC1	Acetyl-CoA carboxylase 1
FASN	Fatty Acid Synthase
SREBP	Sterol regulatory element-binding protein
LDLR	Low density lipoprotein receptor
ACAT-1	Acyl-Coenzyme A: Cholesterol Acyltransferase 1
TIP47	Tail-interacting protein of 47 kDa
PD1	Programmed cell death-1
LAMP1	Lysosomal-associated membrane protein 1
CD107a	Cluster of Differentiation 107a
Treg	Regulatory T cells
Th 17 cells	T-helper 17 cells.

## References

[1] Feitelson M.A., Arzumanyan A., Kulathinal R.J., Blain S.W., Holcombe R.F., Mahajna J., Marino M., Martinez-Chantar M.L., Nawroth R., Sanchez-Garcia I. (2015). Sustained proliferation in cancer: Mechanisms and novel therapeutic targets. Semin. Cancer Biol..

[2] Hanahan D., Weinberg R.A. (2011). Hallmarks of cancer: The next generation. Cell.

[3] Vogelstein B., Kinzler K.W. (2004). Cancer genes and the pathways they control. Nat. Med..

[4] Wu S., Zhu W., Thompson P., Hannun Y.A. (2018). Evaluating intrinsic and non-intrinsic cancer risk factors. Nat. Commun..

[5] Siegel R.L., Miller K.D., Jemal A. (2020). Cancer statistics, 2020. CA Cancer J. Clin..

[6] Bray F., Jemal A., Grey N., Ferlay J., Forman D. (2012). Global cancer transitions according to the Human Development Index (2008–2030): A population-based study. Lancet Oncol..

[7] Safarzadeh E., Sandoghchian S.S., Baradaran B. (2014). Herbal medicine as inducers of apoptosis in cancer treatment. Adv. Pharm. Bull..

[8] Oyebode O., Kandala N.B., Chilton P.J., Lilford R.J. (2016). Use of traditional medicine in middle-income countries: A WHO-SAGE study. Health Policy Plan..

[9] Nerurkar P., Ray R.B. (2010). Bitter melon: Antagonist to cancer. Pharm. Res..

[10] Jia S., Shen M., Zhang F., Xie J. (2017). Recent Advances in Momordica charantia: Functional Components and Biological Activities. Int. J. Mol. Sci..

[11] Dandawate P.R., Subramaniam D., Padhye S.B., Anant S. (2016). Bitter melon: A panacea for inflammation and cancer. Chin. J. Nat. Med..

[12] Raina K., Kumar D., Agarwal R. (2016). Promise of bitter melon (Momordica charantia) bioactives in cancer prevention and therapy. Semin. Cancer Biol..

[13] Fang E.F., Froetscher L., Scheibye-Knudsen M., Bohr V.A., Wong J.H., Ng T.B. (2019). Emerging Antitumor Activities of the Bitter Melon (*Momordica charantia*). Curr. Protein Pept. Sci..

[14] Farooqi A.A., Khalid S., Tahir F., Sabitaliyevich U.Y., Yaylim I., Attar R., Xu B. (2018). Bitter gourd (*Momordica charantia*) as a rich source of bioactive components to combat cancer naturally: Are we on the right track to fully unlock its potential as inhibitor of deregulated signaling pathways. Food Chem. Toxicol..

[15] Shodehinde S.A., Adefegha S.A., Oboh G., Oyeleye S.I., Olasehinde T.A., Nwanna E.E., Adedayo B.C., Boligon A.A. (2016). Phenolic Composition and Evaluation of Methanol and Aqueous Extracts of Bitter Gourd (*Momordica charantia* L.) Leaves on Angiotensin-I-Converting Enzyme and Some Pro-oxidant-Induced Lipid Peroxidation In Vitro. J. Evid.-Based Complement. Altern. Med..

[16] Tan S., Parks S., Stathopoulos C., Roach P. (2014). Extraction of Flavonoids from Bitter Melon. Food Nutr. Sci..

[17] Haque M., Alam M.D.B., Hossain S. (2011). The efficacy of Cucurbitane type triterpenoids, glycosides and phenolic compounds isolated from Momordica charantia: A review. Int. J. Pharm. Sci. Res..

[18] Qian S., Sun L., Li J., Wu J., Hu G., Han Y., Yu K., Zhang S. (2016). MAP30 inhibits autophagy through enhancing acetyltransferase p300 and induces apoptosis in acute myeloid leukemia cells. Oncol. Rep..

[19] Soundararajan R., Prabha P., Rai U., Dixit A. (2012). Antileukemic Potential of Momordica charantia Seed Extracts on Human Myeloid Leukemic HL60 Cells. Evid.-Based Complement. Altern. Med..

[20] Kobori M., Ohnishi-Kameyama M., Akimoto Y., Yukizaki C., Yoshida M. (2008). Alpha-eleostearic acid and its dihydroxy derivative are major apoptosis-inducing components of bitter gourd. J. Agric. Food Chem..

[21] Kai H., Akamatsu E., Torii E., Kodama H., Yukizaki C., Sakakibara Y., Suiko M., Morishita K., Kataoka H., Matsuno K. (2011). Inhibition of proliferation by agricultural plant extracts in seven human adult T-cell leukaemia (ATL)-related cell lines. J. Nat. Med..

[22] Jilka C., Strifler B., Fortner G.W., Hays E.F., Takemoto D.J. (1983). In vivo antitumor activity of the bitter melon (*Momordica charantia*). Cancer Res..

[23] Jiang Y., Miao J., Wang D., Zhou J., Liu B., Jiao F., Liang J., Wang Y., Fan C., Zhang Q. (2018). MAP30 promotes apoptosis of U251 and U87 cells by suppressing the LGR5 and Wnt/beta-catenin signaling pathway, and enhancing Smac expression. Oncol. Lett..

[24] Manoharan G., Jaiswal S.R., Singh J. (2014). Effect of alpha, beta momorcharin on viability, caspase activity, cytochrome c release and on cytosolic calcium levels in different cancer cell lines. Mol. Cell. Biochem..

[25] Wang X., Sun W., Cao J., Qu H., Bi X., Zhao Y. (2012). Structures of new triterpenoids and cytotoxicity activities of the isolated major compounds from the fruit of *Momordica charantia* L.. J. Agric. Food Chem..

[26] Hsiao P.C., Liaw C.C., Hwang S.Y., Cheng H.L., Zhang L.J., Shen C.C., Hsu F.L., Kuo Y.H. (2013). Antiproliferative and hypoglycemic cucurbitane-type glycosides from the fruits of Momordica charantia. J. Agric. Food Chem..

[27] Ray R.B., Raychoudhuri A., Steele R., Nerurkar P. (2010). Bitter melon (*Momordica charantia*) extract inhibits breast cancer cell proliferation by modulating cell cycle regulatory genes and promotes apoptosis. Cancer Res..

[28] Muhammad N., Steele R., Isbell T.S., Philips N., Ray R.B. (2017). Bitter melon extract inhibits breast cancer growth in preclinical model by inducing autophagic cell death. Oncotarget.

[29] Shim S.H., Sur S., Steele R., Albert C.J., Huang C., Ford D.A., Ray R.B. (2018). Disrupting cholesterol esterification by bitter melon suppresses triple-negative breast cancer cell growth. Mol. Carcinog..

[30] Bai L.Y., Chiu C.F., Chu P.C., Lin W.Y., Chiu S.J., Weng J.R. (2016). A triterpenoid from wild bitter gourd inhibits breast cancer cells. Sci. Rep..

[31] Weng J.R., Bai L.Y., Chiu C.F., Hu J.L., Chiu S.J., Wu C.Y. (2013). Cucurbitane Triterpenoid from Momordica charantia Induces Apoptosis and Autophagy in Breast Cancer Cells, in Part, through Peroxisome Proliferator-Activated Receptor gamma Activation. Evid.-Based Complement. Altern. Med..

[32] Grossmann M.E., Mizuno N.K., Dammen M.L., Schuster T., Ray A., Cleary M.P. (2009). Eleostearic Acid inhibits breast cancer proliferation by means of an oxidation-dependent mechanism. Cancer Prev. Res..

[33] Nagasawa H., Watanabe K., Inatomi H. (2002). Effects of bitter melon (*Momordica charantia* L.) or ginger rhizome (*Zingiber offifinale* rosc) on spontaneous mammary tumorigenesis in SHN mice. Am. J. Chin. Med..

[34] Kwatra D., Subramaniam D., Ramamoorthy P., Standing D., Moran E., Velayutham R., Mitra A., Umar S., Anant S. (2013). Methanolic extracts of bitter melon inhibit colon cancer stem cells by affecting energy homeostasis and autophagy. Evid.-Based Complement. Altern. Med..

[35] Kwatra D., Venugopal A., Standing D., Ponnurangam S., Dhar A., Mitra A., Anant S. (2013). Bitter melon extracts enhance the activity of chemotherapeutic agents through the modulation of multiple drug resistance. J. Pharm. Sci..

[36] Kohno H., Yasui Y., Suzuki R., Hosokawa M., Miyashita K., Tanaka T. (2004). Dietary seed oil rich in conjugated linolenic acid from bitter melon inhibits azoxymethane-induced rat colon carcinogenesis through elevation of colonic PPARgamma expression and alteration of lipid composition. Int. J. Cancer.

[37] Konishi T., Satsu H., Hatsugai Y., Aizawa K., Inakuma T., Nagata S., Sakuda S.H., Nagasawa H., Shimizu M. (2004). Inhibitory effect of a bitter melon extract on the P-glycoprotein activity in intestinal Caco-2 cells. Br. J. Pharmacol..

[38] Yasui Y., Hosokawa M., Sahara T., Suzuki R., Ohgiya S., Kohno H., Tanaka T., Miyashita K. (2005). Bitter gourd seed fatty acid rich in 9c,11t,13t-conjugated linolenic acid induces apoptosis and up-regulates the GADD45, p53 and PPARgamma in human colon cancer Caco-2 cells. Prostaglandins Leukot. Essent. Fat. Acids.

[39] Chipps E.S., Jayini R., Ando S., Protzman A.D., Muhi M.Z., Mottaleb M.A., Malkawi A., Islam M.R. (2012). Cytotoxicity analysis of active components in bitter melon (*Momordica charantia*) seed extracts using human embryonic kidney and colon tumor cells. Nat. Prod. Commun..

[40] Sur S., Nakanishi H., Flaveny C., Ippolito J.E., McHowat J., Ford D.A., Ray R.B. (2019). Inhibition of the key metabolic pathways, glycolysis and lipogenesis, of oral cancer by bitter melon extract. Cell Commun. Signal. CCS.

[41] Sur S., Steele R., Aurora R., Varvares M., Schwetye K.E., Ray R.B. (2018). Bitter Melon Prevents the Development of 4-NQO-Induced Oral Squamous Cell Carcinoma in an Immunocompetent Mouse Model by Modulating Immune Signaling. Cancer Prev. Res..

[42] Bhattacharya S., Muhammad N., Steele R., Kornbluth J., Ray R.B. (2017). Bitter Melon Enhances Natural Killer-Mediated Toxicity against Head and Neck Cancer Cells. Cancer Prev. Res..

[43] Bhattacharya S., Muhammad N., Steele R., Peng G., Ray R.B. (2016). Immunomodulatory role of bitter melon extract in inhibition of head and neck squamous cell carcinoma growth. Oncotarget.

[44] Rajamoorthi A., Shrivastava S., Steele R., Nerurkar P., Gonzalez J.G., Crawford S., Varvares M., Ray R.B. (2013). Bitter melon reduces head and neck squamous cell carcinoma growth by targeting c-Met signaling. PLoS ONE.

[45] Brennan V.C., Wang C.M., Yang W.H. (2012). Bitter melon (*Momordica charantia*) extract suppresses adrenocortical cancer cell proliferation through modulation of the apoptotic pathway, steroidogenesis, and insulin-like growth factor type 1 receptor/RAC-alpha serine/threonine-protein kinase signaling. J. Med. Food.

[46] Yue J., Sun Y., Xu J., Cao J., Chen G., Zhang H., Zhang X., Zhao Y. (2019). Cucurbitane triterpenoids from the fruit of Momordica charantia L. and their anti-hepatic fibrosis and anti-hepatoma activities. Phytochemistry.

[47] Fang E.F., Zhang C.Z., Wong J.H., Shen J.Y., Li C.H., Ng T.B. (2012). The MAP30 protein from bitter gourd (*Momordica charantia*) seeds promotes apoptosis in liver cancer cells in vitro and in vivo. Cancer Lett..

[48] Ali M.M., Borai I.H., Ghanem H.M., Abdel-Halim A.H., Mousa F.M. (2018). The prophylactic and therapeutic effects of Momordica charantia methanol extract through controlling different hallmarks of the hepatocarcinogenesis. Biomed. Pharmacother..

[49] Nerurkar P.V., Pearson L., Efird J.T., Adeli K., Theriault A.G., Nerurkar V.R. (2005). Microsomal triglyceride transfer protein gene expression and ApoB secretion are inhibited by bitter melon in HepG2 cells. J. Nutr..

[50] Thiagarajan S., Arapoc D.J., Husna Shafie N., Keong Y.Y., Bahari H., Adam Z., Ei T. (2019). Momordica charantia (Indian and Chinese Bitter Melon) Extracts Inducing Apoptosis in Human Lung Cancer Cell Line A549 via ROS-Mediated Mitochodria Injury. Evid.-Based Complement. Altern. Med..

[51] Fan X., He L., Meng Y., Li G., Li L., Meng Y. (2015). Alpha-MMC and MAP30, two ribosome-inactivating proteins extracted from Momordica charantia, induce cell cycle arrest and apoptosis in A549 human lung carcinoma cells. Mol. Med. Rep..

[52] Hsu H.Y., Lin J.H., Li C.J., Tsang S.F., Tsai C.H., Chyuan J.H., Chiu S.J., Chuang S.E. (2012). Antimigratory Effects of the Methanol Extract from Momordica charantia on Human Lung Adenocarcinoma CL1 Cells. Evid.-Based Complement. Altern. Med..

[53] Pitchakarn P., Umsumarng S., Mapoung S., Ting P., Temviriyanukul P., Punfa W., Pompimon W., Limtrakul P. (2017). Kuguacin J isolated from bitter melon leaves modulates paclitaxel sensitivity in drug-resistant human ovarian cancer cells. J. Nat. Med..

[54] Yung M.M., Ross F.A., Hardie D.G., Leung T.H., Zhan J., Ngan H.Y., Chan D.W. (2016). Bitter Melon (*Momordica charantia*) Extract Inhibits Tumorigenicity and Overcomes Cisplatin-Resistance in Ovarian Cancer Cells Through Targeting AMPK Signaling Cascade. Integr. Cancer Ther..

[55] Kaur M., Deep G., Jain A.K., Raina K., Agarwal C., Wempe M.F., Agarwal R. (2013). Bitter melon juice activates cellular energy sensor AMP-activated protein kinase causing apoptotic death of human pancreatic carcinoma cells. Carcinogenesis.

[56] Somasagara R.R., Deep G., Shrotriya S., Patel M., Agarwal C., Agarwal R. (2015). Bitter melon juice targets molecular mechanisms underlying gemcitabine resistance in pancreatic cancer cells. Int. J. Oncol..

[57] Dhar D., Deep G., Kumar S., Wempe M.F., Raina K., Agarwal C., Agarwal R. (2018). Bitter melon juice exerts its efficacy against pancreatic cancer via targeting both bulk and cancer stem cells. Mol. Carcinog..

[58] Dhar D., Raina K., Kant R., Wempe M.F., Serkova N.J., Agarwal C., Agarwal R. (2019). Bitter melon juice-intake modulates glucose metabolism and lactate efflux in tumors in its efficacy against pancreatic cancer. Carcinogenesis.

[59] Pitchakarn P., Suzuki S., Ogawa K., Pompimon W., Takahashi S., Asamoto M., Limtrakul P., Shirai T. (2012). Kuguacin J, a triterpeniod from Momordica charantia leaf, modulates the progression of androgen-independent human prostate cancer cell line, PC3. Food Chem. Toxicol..

[60] Pitchakarn P., Ogawa K., Suzuki S., Takahashi S., Asamoto M., Chewonarin T., Limtrakul P., Shirai T. (2010). Momordica charantia leaf extract suppresses rat prostate cancer progression in vitro and in vivo. Cancer Sci..

[61] Ru P., Steele R., Nerurkar P.V., Phillips N., Ray R.B. (2011). Bitter melon extract impairs prostate cancer cell-cycle progression and delays prostatic intraepithelial neoplasia in TRAMP model. Cancer Prev. Res..

[62] Xiong S.D., Yu K., Liu X.H., Yin L.H., Kirschenbaum A., Yao S., Narla G., DiFeo A., Wu J.B., Yuan Y. (2009). Ribosome-inactivating proteins isolated from dietary bitter melon induce apoptosis and inhibit histone deacetylase-1 selectively in premalignant and malignant prostate cancer cells. Int. J. Cancer.

[63] Agrawal R.C., Beohar T. (2010). Chemopreventive and anticarcinogenic effects of Momordica charantia extract. Asian Pac. J. Cancer Prev..

[64] Akihisa T., Higo N., Tokuda H., Ukiya M., Akazawa H., Tochigi Y., Kimura Y., Suzuki T., Nishino H. (2007). Cucurbitane-type triterpenoids from the fruits of Momordica charantia and their cancer chemopreventive effects. J. Nat. Prod..

[65] Ganguly C., De S., Das S. (2000). Prevention of carcinogen-induced mouse skin papilloma by whole fruit aqueous extract of *Momordica charantia*. Eur. J. Cancer prevention Off. J. Eur. Cancer Prev. Organ..

[66] Deep G., Dasgupta T., Rao A.R., Kale R.K. (2004). Cancer preventive potential of *Momordica charantia* L. against benzo(a)pyrene induced fore-stomach tumourigenesis in murine model system. Indian J. Exp. Biol..

[67] Li Y., Yin L., Zheng L., Xu L., Xu Y., Zhao Y., Qi Y., Yao J., Han X., Liu K. (2012). Application of high-speed counter-current chromatography coupled with a reverse micelle solvent system to separate three proteins from Momordica charantia. J. Chromatogr. B Anal. Technol. Biomed. Life Sci..

[68] Li C.J., Tsang S.F., Tsai C.H., Tsai H.Y., Chyuan J.H., Hsu H.Y. (2012). Momordica charantia Extract Induces Apoptosis in Human Cancer Cells through Caspase- and Mitochondria-Dependent Pathways. Evid.-Based Complement. Altern. Med..

[69] Pitchakarn P., Ohnuma S., Pintha K., Pompimon W., Ambudkar S.V., Limtrakul P. (2012). Kuguacin J isolated from Momordica charantia leaves inhibits P-glycoprotein (ABCB1)-mediated multidrug resistance. J. Nutr. Biochem..

[70] Lee-Huang S., Huang P.L., Sun Y., Chen H.C., Kung H.F., Huang P.L., Murphy W.J. (2000). Inhibition of MDA-MB-231 human breast tumor xenografts and HER2 expression by anti-tumor agents GAP31 and MAP30. Anticancer Res..

[71] Konishi T., Satsu H., Hatsugai Y., Aizawa K., Inakuma T., Nagata S., Sakuda S.H., Nagasawa H., Shimizu M. (2004). A bitter melon extract inhibits the P-glycoprotein activity in intestinal Caco-2 cells: Monoglyceride as an active compound. BioFactors.

[72] Claflin A.J., Vesely D.L., Hudson J.L., Bagwell C.B., Lehotay D.C., Lo T.M., Fletcher M.A., Block N.L., Levey G.S. (1978). Inhibition of growth and guanylate cyclase activity of an undifferentiated prostate adenocarcinoma by an extract of the balsam pear (*Momordica charantia* abbreviata). Proc. Natl. Acad. Sci. USA.

[73] Lin J.Y., Hou M.J., Chen Y.C. (1978). Isolation of toxic and non-toxic lectins from the bitter pear melon Momordica charantia Linn. Toxicon Off. J. Int. Soc. Toxinol..

[74] Lu K.H., Tseng H.C., Liu C.T., Huang C.J., Chyuan J.H., Sheen L.Y. (2014). Wild bitter gourd protects against alcoholic fatty liver in mice by attenuating oxidative stress and inflammatory responses. Food Funct..

[75] Fang E.F., Zhang C.Z., Fong W.P., Ng T.B. (2012). RNase MC2: A new Momordica charantia ribonuclease that induces apoptosis in breast cancer cells associated with activation of MAPKs and induction of caspase pathways. Apoptosis Int. J. Program. Cell Death.

[76] Dia V.P., Krishnan H.B. (2016). BG-4, a novel anticancer peptide from bitter gourd (Momordica charantia), promotes apoptosis in human colon cancer cells. Sci. Rep..

[77] Pan W.L., Wong J.H., Fang E.F., Chan Y.S., Ng T.B., Cheung R.C. (2014). Preferential cytotoxicity of the type I ribosome inactivating protein alpha-momorcharin on human nasopharyngeal carcinoma cells under normoxia and hypoxia. Biochem. Pharmacol..

[78] Qian Q., Chen W., Cao Y., Cao Q., Cui Y., Li Y., Wu J. (2019). Targeting Reactive Oxygen Species in Cancer via Chinese Herbal Medicine. Oxidative Med. Cell. Longev..

[79] Landskron G., De la Fuente M., Thuwajit P., Thuwajit C., Hermoso M.A. (2014). Chronic inflammation and cytokines in the tumor microenvironment. J. Immunol. Res..

[80] Pathania S., Bhatia R., Baldi A., Singh R., Rawal R.K. (2018). Drug metabolizing enzymes and their inhibitors’ role in cancer resistance. Biomed. Pharmacother..

[81] Younus H. (2018). Therapeutic potentials of superoxide dismutase. Int. J. Health Sci..

[82] Visconti R., Della Monica R., Grieco D. (2016). Cell cycle checkpoint in cancer: A therapeutically targetable double-edged sword. J. Exp. Clin. Cancer Res..

[83] Martin G.S. (2003). Cell signaling and cancer. Cancer Cell.

[84] Zhang Y., Xia M., Jin K., Wang S., Wei H., Fan C., Wu Y., Li X., Li X., Li G. (2018). Function of the c-Met receptor tyrosine kinase in carcinogenesis and associated therapeutic opportunities. Mol. Cancer.

[85] Iida M., Harari P.M., Wheeler D.L., Toulany M. (2020). Targeting AKT/PKB to improve treatment outcomes for solid tumors. Mutat. Res..

[86] Fresno Vara J.A., Casado E., de Castro J., Cejas P., Belda-Iniesta C., Gonzalez-Baron M. (2004). PI3K/Akt signalling pathway and cancer. Cancer Treat. Rev..

[87] Chen Q., Kang J., Fu C. (2018). The independence of and associations among apoptosis, autophagy, and necrosis. Signal. Transduct. Target. Ther..

[88] Ferreira C.G., Epping M., Kruyt F.A., Giaccone G. (2002). Apoptosis: Target of cancer therapy. Clin. Cancer Res. Off. J. Am. Assoc. Cancer Res..

[89] White E. (2012). Deconvoluting the context-dependent role for autophagy in cancer. Nat. Rev. Cancer.

[90] Desai A., Yan Y., Gerson S.L. (2019). Concise Reviews: Cancer Stem Cell Targeted Therapies: Toward Clinical Success. Stem Cells Transl. Med..

[91] Szafarowski T., Szczepanski M.J. (2014). Cancer stem cells in head and neck squamous cell carcinoma. Otolaryngol. Pol..

[92] Liskova A., Kubatka P., Samec M., Zubor P., Mlyncek M., Bielik T., Samuel S.M., Zulli A., Kwon T.K., Busselberg D. (2019). Dietary Phytochemicals Targeting Cancer Stem Cells. Molecules.

[93] Markowitz J., Carson W.E. (2013). Review of S100A9 biology and its role in cancer. Biochim. Biophys. Acta.

[94] Economopoulou P., Perisanidis C., Giotakis E.I., Psyrri A. (2016). The emerging role of immunotherapy in head and neck squamous cell carcinoma (HNSCC): Anti-tumor immunity and clinical applications. Ann. Transl. Med..

[95] Bhatia A., Burtness B. (2017). Novel Molecular Targets for Chemoprevention in Malignancies of the Head and Neck. Cancers.

[96] Oliveira I., Nunes A., Lima A., Borralho P., Rodrigues C., Ferreira R.B., Ribeiro A.C. (2019). New Lectins from Mediterranean Flora. Activity against HT29 Colon Cancer Cells. Int. J. Mol. Sci..

[97] Lekka M., Laidler P., Labedz M., Kulik A.J., Lekki J., Zajac W., Stachura Z. (2006). Specific detection of glycans on a plasma membrane of living cells with atomic force microscopy. Chem. Biol..

[98] Fang E.F., Zhang C.Z., Ng T.B., Wong J.H., Pan W.L., Ye X.J., Chan Y.S., Fong W.P. (2012). Momordica Charantia lectin, a type II ribosome inactivating protein, exhibits antitumor activity toward human nasopharyngeal carcinoma cells in vitro and in vivo. Cancer Prev. Res..

[99] Park J.I., Bae H.R., Kim C.G., Stonik V.A., Kwak J.Y. (2014). Relationships between chemical structures and functions of triterpene glycosides isolated from sea cucumbers. Front. Chem..

[100] Koczurkiewicz P., Czyz J., Podolak I., Wojcik K., Galanty A., Janeczko Z., Michalik M. (2015). Multidirectional effects of triterpene saponins on cancer cells—Mini-review of in vitro studies. Acta Biochim. Pol..

[101] Sak K. (2014). Cytotoxicity of dietary flavonoids on different human cancer types. Pharmacogn. Rev..

[102] Meng Y., Lin S., Liu S., Fan X., Li G., Meng Y. (2014). A novel method for simultaneous production of two ribosome-inactivating proteins, alpha-MMC and MAP30, from *Momordica charantia* L.. PLoS ONE.

[103] Takemoto D.J., Jilka C., Rockenbach S., Hughes J.V. (1983). Purification and characterization of a cytostatic factor with anti-viral activity from the bitter melon. Prep. Biochem..

[104] Panche A.N., Diwan A.D., Chandra S.R. (2016). Flavonoids: An overview. J. Nutr. Sci..

[105] Kanakis C.D., Tarantilis P.A., Polissiou M.G., Diamantoglou S., Tajmir-Riahi H.A. (2007). An overview of DNA and RNA bindings to antioxidant flavonoids. Cell Biochem. Biophys..

[106] Kanwal R., Gupta S. (2012). Epigenetic modifications in cancer. Clin. Genet..

[107] Thakur V.S., Deb G., Babcook M.A., Gupta S. (2014). Plant phytochemicals as epigenetic modulators: Role in cancer chemoprevention. AAPS J..

[108] Busch C., Burkard M., Leischner C., Lauer U.M., Frank J., Venturelli S. (2015). Epigenetic activities of flavonoids in the prevention and treatment of cancer. Clin. Epigenetics.

[109] Sung H.C., Liu C.W., Hsiao C.Y., Lin S.R., Yu I.S., Lin S.W., Chiang M.H., Liang C.J., Pu C.M., Chen Y.C. (2018). The effects of wild bitter gourd fruit extracts on ICAM-1 expression in pulmonary epithelial cells of C57BL/6J mice and microRNA-221/222 knockout mice: Involvement of the miR-221/-222/PI3K/AKT/NF-kappaB pathway. Phytomed. Int. J. Phytother. Phytopharm..

